# Molecular epidemiological characteristics of *Brucella* in Guizhou Province, China, from 2009 to 2021

**DOI:** 10.3389/fmicb.2023.1188469

**Published:** 2023-06-23

**Authors:** Qinqin Tan, Yue Wang, Ying Liu, Zhongfa Tao, Chun Yu, Yan Huang, Xinggui Yang, Xia Ying, Yong Hu, Shijun Li

**Affiliations:** ^1^Laboratory Center, Center for Disease Control and Prevention of Guizhou Provincial, Guiyang, Guizhou, China; ^2^The Key Laboratory of Environmental Pollution Monitoring and Disease Control, Ministry of Education, School of Public Health, University of Guizhou Medical, Guiyang, Guizhou, China; ^3^Center for Disease Control and Prevention of Guizhou Provincial, Institute of Infectious Disease Control, Guiyang, Guizhou, China

**Keywords:** brucellosis, *Brucella*, MLVA, MLST, *rpoB*, Guizhou Province

## Abstract

**Introduction:**

Brucellosis was made statutorily notifiable in 1955, in China, while in Guizhou Province, the pathogen of human brucellosis was isolated for the first time in 2011. However, currently, the brucellosis epidemic is becoming more and more severe in Guizhou Province. The type distribution and genetic characteristics of *Brucella* in Guizhou Province, as well as its evolutionary relationship with domestic and foreign strains, are still unclear.

**Methods:**

MLST, MLVA, and *rpoB* typing techniques were used for the molecular epidemiological study of the 83 *Brucella* isolates in Guizhou province.

**Results:**

Among the 83 *Brucella* strains, MLST identified three ST genotypes, of which ST39 is a newly reported type in China. MLVA-16 generated 49 genotypes, and MLVA-11 generated 5 known genotypes and 2 unreported genotypes. Six genotypes were identified by *rpoB* technology.

**Discussion:**

MLVA has a high resolution, but differences at the Bruce 04 and 16 loci cannot exclude associations between epidemics, and combining MLST and *rpoB* typing methods for epidemiologic tracing can avoid erroneous judgments. Moreover, through the combined analysis of the three typing techniques, the possible origin of the new *Brucella* can be reasonably inferred, which is also conducive to promoting the subsequent research of the novel *Brucella*.

## Introduction

Brucellosis, a zoonotic disease caused by *Brucella* spp., can be transmitted from animal reservoirs, such as cattle, sheep, and goats, to humans through ingestion of un-pasteurized animal products or direct contact with infected animals, placentas, or aborted fetuses (Pappas et al., 2006; Lai et al., 2017). Currently, the disease remains one of the most common bacterial zoonotic diseases in the world (Pappas et al., 2006; Cross et al., 2019; Suárez-Esquivel et al., 2020), with over half a million new cases annually and prevalence rates in some countries exceeding 10 cases per 100,000 population (Franco et al., 2007). In China, human brucellosis was first recorded in 1905 (Deqiu et al., 2002; Lai et al., 2017). In 1955, brucellosis was made statutorily notifiable in China (Lai et al., 2017). At present, brucellosis is endemic in 31 provinces or autonomous regions in China, most of which are prevalent in northern China, where people are economically dependent on ruminant livestock (Jiang et al., 2011). Meanwhile, *B. melitensis* and *B. abortus* are the main pathogenic bacteria of brucellosis epidemics in China (Tian et al., 2017b).

Brucellosis is prevalent in the northern pastoral provinces of China (Zhong et al., 2013; Lai et al., 2017; Jiang et al., 2020), whereas Guizhou, a non-pastoral province with a predominantly highland mountainous terrain, did not confirm the existence of human brucellosis epidemic etiologically until 2011 (Li et al., 2012). *Brucella* was first isolated from a goat in 2010 (Li et al., 2011) and a patient in 2011 (Li et al., 2012), and then the brucellosis epidemic in Guizhou Province spread rapidly, and sporadic and cluster epidemics have occurred frequently. By 2015, the brucellosis epidemic had covered nine cities and prefectures in Guizhou province, with only some counties not reported (Jiang et al., 2017). As brucellosis is an important zoonotic infection in Guizhou Province in recent years, currently, the prevention and control situation of brucellosis is becoming increasingly serious here. In addition, the epidemic situation of human brucellosis and the molecular characteristics of *Brucella* remain unclear. The genetic relationships among *Brucella* isolated from different hosts, different regions, and at different times need to be clarified. In addition, the evolutionary relationship among *Brucella* from Guizhou Province and other provinces in China and other countries remains to be revealed.

To answer these questions, several techniques are employed for analysis. The classical bio-typing methods cannot analyze the correlation among the *Brucella*, while most typing tools lack sufficient discriminatory power. To overcome these drawbacks, *Brucella* multi-locus sequence analysis (MLST) and multi-locus variable number tandem repeat analysis (MLVA) have been proposed as complementary technical approaches to classical bio-typing methods. MLST, a molecular typing method for discovering bacterial variants by determining the nucleotide sequences of multiple housekeeping genes of study subjects, with clear typing results and a Shared Database, is used for bacterial typing and genetic characterization studies (Whatmore et al., 2007; Cao et al., 2018b). MLVA, based on its high discriminatory power of a multiple-locus variable number tandem repeat (VNTR) and a shared database of genotype information, facilitates the tracking analysis of brucellosis outbreaks (Al Dahouk et al., 2007; Ma et al., 2016; Wareth et al., 2020). In recent years, genome sequencing provides a new platform for the study of bacteria. The single nucleotide polymorphism analysis of the *rpoB* (β subunit of RNA polymerase) gene region can be used not only for the *Brucella* species (types) identification but also for *Brucella* molecular epidemiological investigation studies (Marianelli et al., 2006; Tan et al., 2017).

The current study aimed to reveal the prevalence of human brucellosis in Guizhou Province in recent years and also to explore the molecular characteristics of Brucella isolates by combining MLVA, MLST, and *rpoB* genotyping techniques. The results of this study contribute to our understanding of the main transmission routes associated with this pathogen as well as provide a scientific basis for the prevention and control of brucellosis in Guizhou Province.

## Materials and methods

### Epidemiological data collection

Data on human brucellosis cases in Guizhou Province were collected from the online National Notifiable Infectious Disease Reporting Information System of the Chinese Center for Disease Control and Prevention for the period 2009–2021. Subsequently, statistical methods were used to analyze the regional distribution and population distribution characteristics of human brucellosis in Guizhou Province.

### *Brucella* strains' information

A total of 83 *Brucella* strains were isolated from the nine cities/prefectures of Guizhou Province from 2009 to 2021. The species and biotypes of the 83 isolates have been characterized according to the conventional bio-typing tests, *Brucella-*specific surface proteins 31-PCR (BCSP31-PCR), and AMSO-PCR by using the procedures recommended by WS 269-2019 guidelines of China. The 83 isolates were defined as 81 *B. melitensis* (80 *B. melitensis* bv3 and 1 unspecified biotype), and 2 *B. abortus* bv3 (Table 1). The host origin of 83 isolates shows that four *B. melitensis* bv3 were isolated from goats, and the rest were isolated from humans (Supplementary Table S1).

**Table 1:** Basic information about the 83 *Brucella* strains in Guizhou Province.

**Prefecture**	**No. of strains**	**Bio-type**
Anshun	1	*B. melitensis* bv3
Bijie	8	*B. melitensis* bv3
Guiyang	1	*B. melitensis* bv3
	2	*B. abortus* bv3
Liupanshui	2	*B. melitensis* bv3
Qiandongnan Miao and Dong Autonomous Prefecture	1	*B. melitensis*
	13	*B. melitensis* bv3
Qiannan Buyi and Miao Autonomous Prefecture	15	*B. melitensis* bv3
Qianxinan Buyi and Miao Autonomous Prefecture	9	*B. melitensis* bv3
Tongren	5	*B. melitensis* bv3
Zunyi	26	*B. melitensis* bv3

### Genomic DNA preparation

A total of 83 *Brucella* strains were resuscitated on *Brucella* agar plates and incubated at 37°C for 24–48 h. Then, the genomic DNA of *Brucella*, including the isolates and vaccine strains M5, A19, and S2 were extracted following the boiling lysis method mentioned in the national standard of “Diagnosis of Brucellosis” WS 269-2019.

### MLST genotyping

It has been performed concerning the previously described method (Zhuang, 2015). The program of nine genomes was designed according to Whatmore et al., including 7 housekeeping genes, 1 outer membrane protein gene, and 1 intergenic fragment (Whatmore et al., 2007). The primer sequences of the MLST nine loci are shown in Table 2. Genomic DNA of vaccine strains, including *B. melitensis* M5, *B. abortus* A19, and *B. suis* S2, were used as positive controls to monitor PCR protocol. The PCR cycling parameters of MLST were as follows: 95°C for 5 min, followed by 30 cycles of 94°C for the 30 s, 63°C for 30 s, 72°C for 60 s, and an elongation at 72°C for 10 min. Then, the products were sent to the Biotechnology company for sequencing. Each gene sequence was entered into the MLST online database (https://pubmlst.org/bigsdb?db=pubmlst_brucella_seqdef&page=batchSequenceQuery) to specify its defined value. Subsequently, the profile of the nine genes was identified as a specific sequence type (ST) based on MLST online database. The ST genotypes of the *Brucella* were marked on the map of Guizhou Province for analysis of their distribution characteristics. The Web-based MLST database [https://pubmlst.org/bigsdb?db=pubmlst_brucella_seqdef&page=Download alleles (pubmlst.org)] was utilized to collect MLST genotypes of *Brucella* from different countries. A minimum spanning tree (MST) was constructed using BioNumerics software (version 8.0) based on ST data and an unweighted arithmetic mean algorithm (UPGMA) to show genetic relationships between 83 *Brucella* isolates and representative strains of *Brucella* from China and other countries.

**Table 2:** The primers sequence of *Brucella* MLST, MLVA-16, and *rpoB* genotyping schemes.

**Title**	**Gene names**	**Forward primers (5^′^−3^′^)**	**Reverse primers (5^′^−3^′^)**
MLST	*TrpE*	TATCGGTCGCCGCCAGATCG	GCGTGAAGGCTTGCCATGC
	*Omp25*	CGCACTCTTAAGTCTCTCG	GAACTTGTAGCCGATGCCG
	*Int-hyp*	CTTCTGTTGCCAGGTGCC	TCCTCGACGCCCAGTTTC
	*GyrB*	AAGCTTGCCGATTGCCAG	TCACATGGCCGCTCCAGC
	*Glk*	TCTGTCATTCTGGCCGTGGC	CGGAAAGCCCGGTTAAGGC
	*Gap*	TACGATCGATGTTGGCTAC	TCGCTCATGCGGCTGGAG
	*DnaK*	ATCATCAACGAGCCGACCG	AGCCGTGGAGAAGGTCTGC
	*AroA*	CGTGATCGAGCCGGTCATGA	CGGCAATCTTCGCCCCCAGC
	*CobQ*	GTGTCGCGTCTGCCAGCG	GCAAACAGGCCATCAATATC
MLVA panel 1	Bruce 06	ATGGGATGTGGTAGGGTAATCG	GCGTGACAATCGACTTTTTGTC
	Bruce 08	ATTATTCGCAGGCTCGTGATTC	ACAGAAGGTTTTCCAGCTCGTC
	Bruce 11	CTGTTGATCTGACCTTGCAACC	CCAGACAACAACCTACGTCCTG
	Bruce 12	CGGTAAATCAATTGTCCCATGA	GCCCAAGTTCAACAGGAGTTTC
	Bruce 42	CATCGCCTCAACTATACCGTCA	ACCGCAAAATTTACGCATCG
	Bruce 43	TCTCAAGCCCGATATGGAGAAT	TATTTTCCGCCTGCCCATAAAC
	Bruce 45	ATCCTTGCCTCTCCCTACCAG	CGGGTAAATATCAATGGCTTGG
	Bruce 55	TCAGGCTGTTTCGTCATGTCTT	AATCTGGCGTTCGAGTTGTTCT
MLVA panel 2	Bruce 04^2B^	TAMRA-CTGACGAAGGGAAGGCAATAAG	CGATCTGGAGATTATCGGGAAG
	Bruce 18^2A^	FAM-TATGTTAGGGCAATAGGGCAGT	GATGGTTGAGAGCATTGTGAAG
	Bruce 21^2A^	ROX-CTCATGCGCAACCAAAACA	GATCTCGTGGTCGATAATCTCATT
	Bruce 07^2B^	FAM-GCTGACGGGGAAGAACATCTAT	ACCCTTTTTCAGTCAAGGCAAA
	Bruce 09^2B^	ROX-GCGGATTCGTTCTTCAGTTATC	GGGAGTATGTTTTGGTTGTACATAG
	Bruce 16^2B^	TAMRA-ACGGGAGTTTTTGTTGCTCAAT	GGCCATGTTTCCGTTGATTTAT
	Bruce 19^2A^	FAM-GACGACCCGGACCATGTCT	ACTTCACCGTAACGTCGTGGAT
	Bruce 30^2B^	TAMRA-TGACCGCAAAACCATATCCTTC	TATGTGCAGAGCTTCATGTTCG
*rpoB*	*rpoB*	ATGGCTCAGACCCATTCTTTC	TTATTCTGC CGCGTCCGGAA

### MLVA genotyping

MLVA scheme was carried out according to the previous study (Al Dahouk et al., 2007), which is based on 16 loci including 8 minisatellite loci (panel 1) and 8 microsatellite loci (panel 2, subdivided into 2A and 2B). The primer sequences of the 16 loci are shown in Table 2. The genomic DNA of vaccine strains was used as positive controls to monitor the PCR protocol and calibrate the capillary electrophoresis platform. For the loci of panel 1 (bruce 06, 08, 11, 12, 42, 43, 45, and 55), PCR amplifications were performed in 20 μL reaction volumes, and the cycling parameters were as follows: 94°C for 3 min, and then 30 cycles of 94°C for 30 s, 60°C for 30 s, and 72°C for 50 s, and elongation at 72°C for 3 min. The products were detected by 1.2% agarose gel electrophoresis, and the differential sequences of each locus product were sent to the Biotechnology company for sequencing. Subsequently, the flanking sequences were removed and the values of the loci repeat sequences were calculated. For the loci of panel 2 (bruce 04, 07, 09, 16, 18, 19, 21, and 30), multiplex PCR combining a multicolor capillary electrophoresis approach was performed (Tan et al., 2022b). Specifically, the 5 ends of each locus forward primers within each reaction system were synthesized with 6-carboxyfluorescein (FAM), 5-carboxy tetramethylrhodamine (TAMRA), and carboxy-X-rhodamine (ROX) fluorescent label, respectively (Table 2). The amplification of the loci was performed in 3 sets of 30 μl reaction volumes (Tan et al., 2022b). The cycling parameters were as follows: 96°C for 3 min, followed by 32 cycles of 94°C for 30 s, 62°C for 60 s, and 72°C for 30 s, and an elongation step at 72°C for 5 min. The products were sent to a Biotechnology company for multicolor capillary electrophoresis, and the loci and values were identified according to the color presented in each graph of each specimen. In this study, fragment sizes were converted to repeat units according to the published allele numbering system (Al Dahouk et al., 2007). Differently, the repeat unit of 3 bp was used to calculate the value of locus Bruce 19 per sample (Sun et al., 2016).

Polymorphisms at each locus of the 83 *Brucella* isolates were quantified using the Hunter and Gaston diversity index (Hunter and Gaston, 1988). Then, the 16 loci profile of *Brucella* strains, including the 83 isolates, vaccine strains, and the representative strains of *Brucella* from different parts of China, were analyzed by BioNumerics software, based on classification coefficients and UPGMA, to clarify the affinities between the strains. Furthermore, The MLVA genotypes of the isolates were marked on the map of Guizhou for analysis of their distribution characteristics. The online database (http://mlva.u-psud.fr/) was used to identify the geographic origin of the 2 *B. abortus* isolates and collect MLVA genotypes from different countries. In the study, a total of 269 *B. melitensis* strains were included for analysis, including the American group (37), West Mediterranean group (29), East Mediterranean group (60), and China group (62). The MST was constructed based on the MLVA-11 profile of 269 *B. melitensis* to study the phylogeographic relationships (Liu et al., 2017).

### The *rpoB* genotyping

The *rpoB* gene was performed using the scheme described previously (Marianelli et al., 2006). The primer sequences of the whole length of the *rpoB* gene with 4,134 bp are shown in Table 2. The genomic DNA of vaccine strains were used as positive controls to monitor PCR protocol. The amplifications were performed in 25 μl reaction volumes, and the cycling parameters were as follows: 94°C for 4 min, followed by 35 cycles of 94°C for 30 s, 60°C for 40 s, and 72°C for 3 min, and elongation at 72°C for 7 min. The products were detected by 1.0% agarose gel electrophoresis, and the products with a target band of 4,134 bp were sent to the Biotechnology company for sequencing.

The *rpoB* gene sequences of *Brucella* isolates were compared with *Brucella* 16M using DNAMAN software to clarify the single nucleotide polymorphism loci of the isolates and determine the *rpoB* genotypes. Then, the geographical distribution of *rpoB* genotypes was analyzed for 83 isolates. Subsequently, a phylogenetic tree was constructed in MEGA software (version 7.0) with the neighbor-joining method among 88 *Brucella* strains, including 83 *Brucella* isolates, vaccine strains, *B. abortus* 2,308 (Gene ID: 3787856), and *B. melitensis* 16M (Gene ID: 29593532).

## Results

### The epidemiologic situation of human brucellosis in Guizhou Province

**Figure 1:**
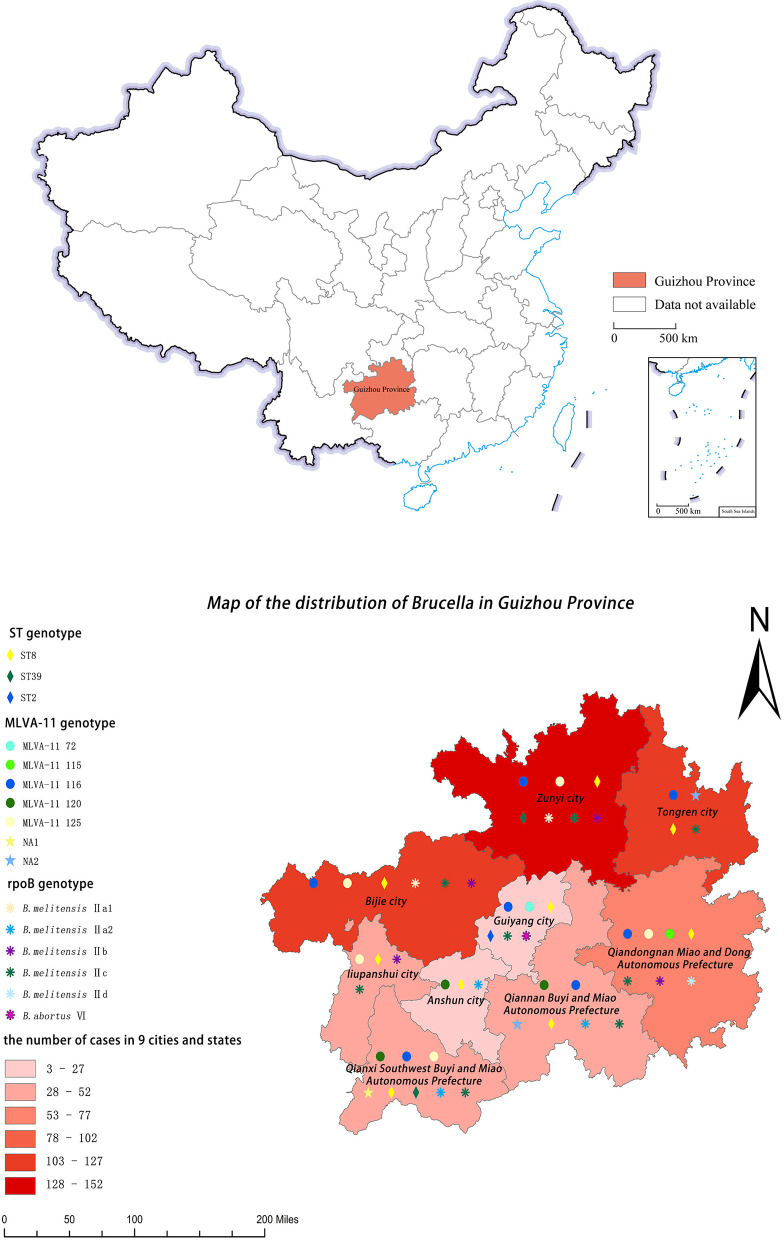
Geographical distribution of the number of human brucellosis and genotype types of *Brucella* isolates in Guizhou Province, 2009–2021. The shade of blocks reflects the number of human cases over the 13 years. The shapes and colors of the icons represent the types of *Brucella*.

**Figure 2:**
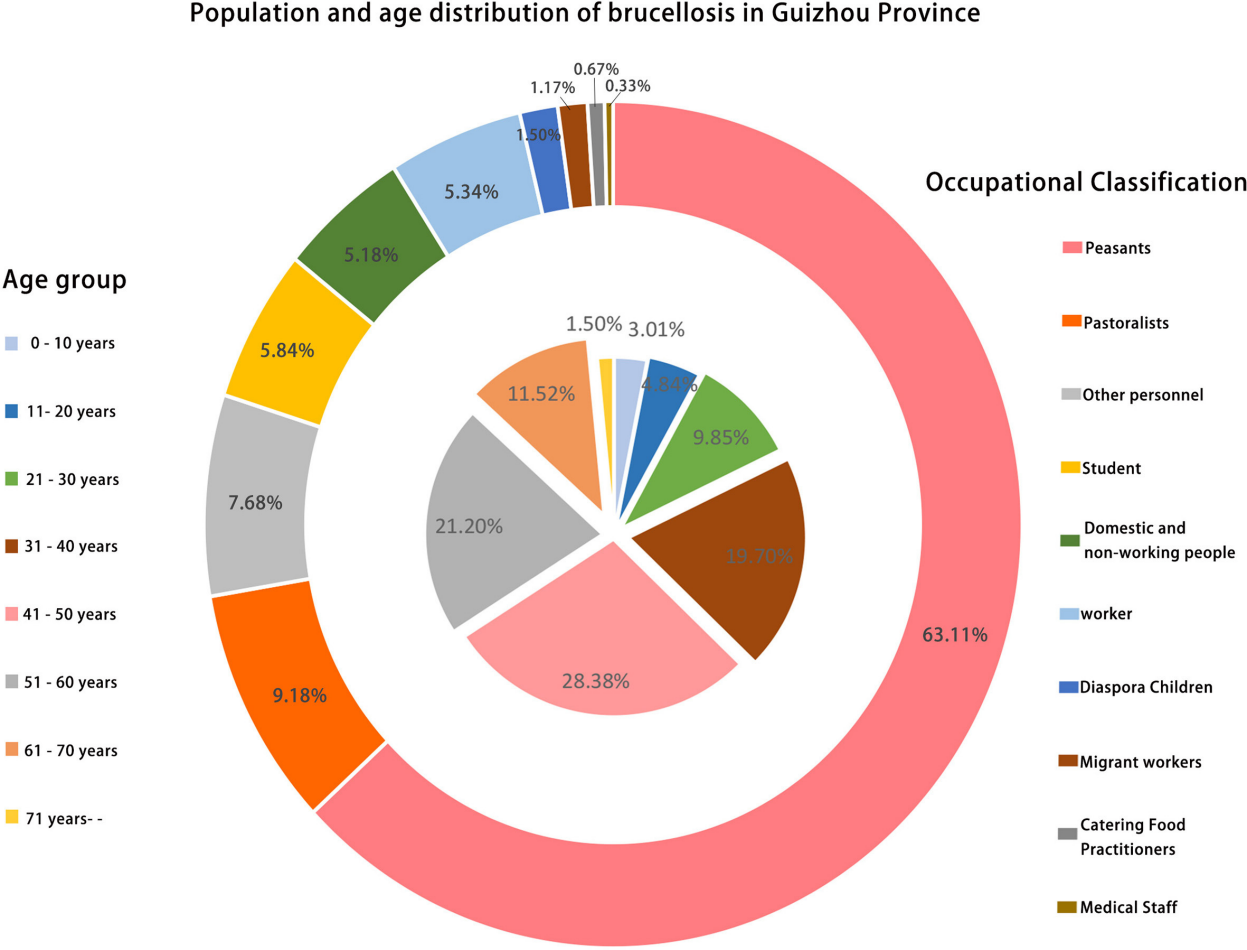
Population distribution characteristics of human brucellosis in Guizhou Province. The pie chart in the middle represents the proportional characteristics of the age distribution of brucellosis patients, and the legend is annotated on the left. The ring in the outer circle represents the proportional characteristics of the occupational distribution of brucellosis patients, and the legend is annotated on the right side.

A total of 599 human brucelloses were reported during 2009–2021, which covered all the cities/prefectures in Guizhou Province, with large cases in Zunyi (Figure 1). The occupation of the patients includes peasants, pastoralists, students, domestic and non-working people, workers, catering food practitioners, etc., with farmers being the main group of brucellosis patients, accounting for 63.11%. The age distribution of the patients showed the largest proportion of 41–50 years old, with 28.38% (Figure 2).

### MLST genotyping results

The 83 *Brucella* isolates, based on 9 loci MLST technology, were 3 known MLST genotypes: ST8 (*N* = 71; 85.54%), ST39 (*N* = 10; 12.05%), and ST2 (*N* = 2; 2.41%). Among the 81 *B. melitensis*, 71 strains were identified as ST8, and the other 10 were ST39. The 2 *B. abortus* were ST2 (Table 3).

**Table 3:** The MLST genotype characteristics of the 83 *Brucella* strains isolates.

**Genotype**	The profile of ST genotype^*^	**Species**	**No. of strains**	**Percentage**
ST8	3-2-3-2-1-5-3-8-2	*B. melitensis*	71	85.54%
ST39	3-2-24-2-1-5-3-8-2	*B. melitensis*	10	12.05%
ST2	2-1-2-2-1-3-1-1-1	*B. abortus*	2	2.41%

^*^The order of the nine gene loci in the MLST profile is *gap, aroA, glk, dnak, gyrB, tryE, cobQ, omp25*, and *Int-hyp*.

The geographical distribution of the ST genotypes showed that the ST8 *Brucella* strains were widely distributed in the 9 cities/states in Guizhou Province, the ST39 were in Zunyi City and Qianxinan Buyi and Miao Autonomous Prefecture, while the ST2 was only in Guiyang City (Figure 1).

**Figure 3:**
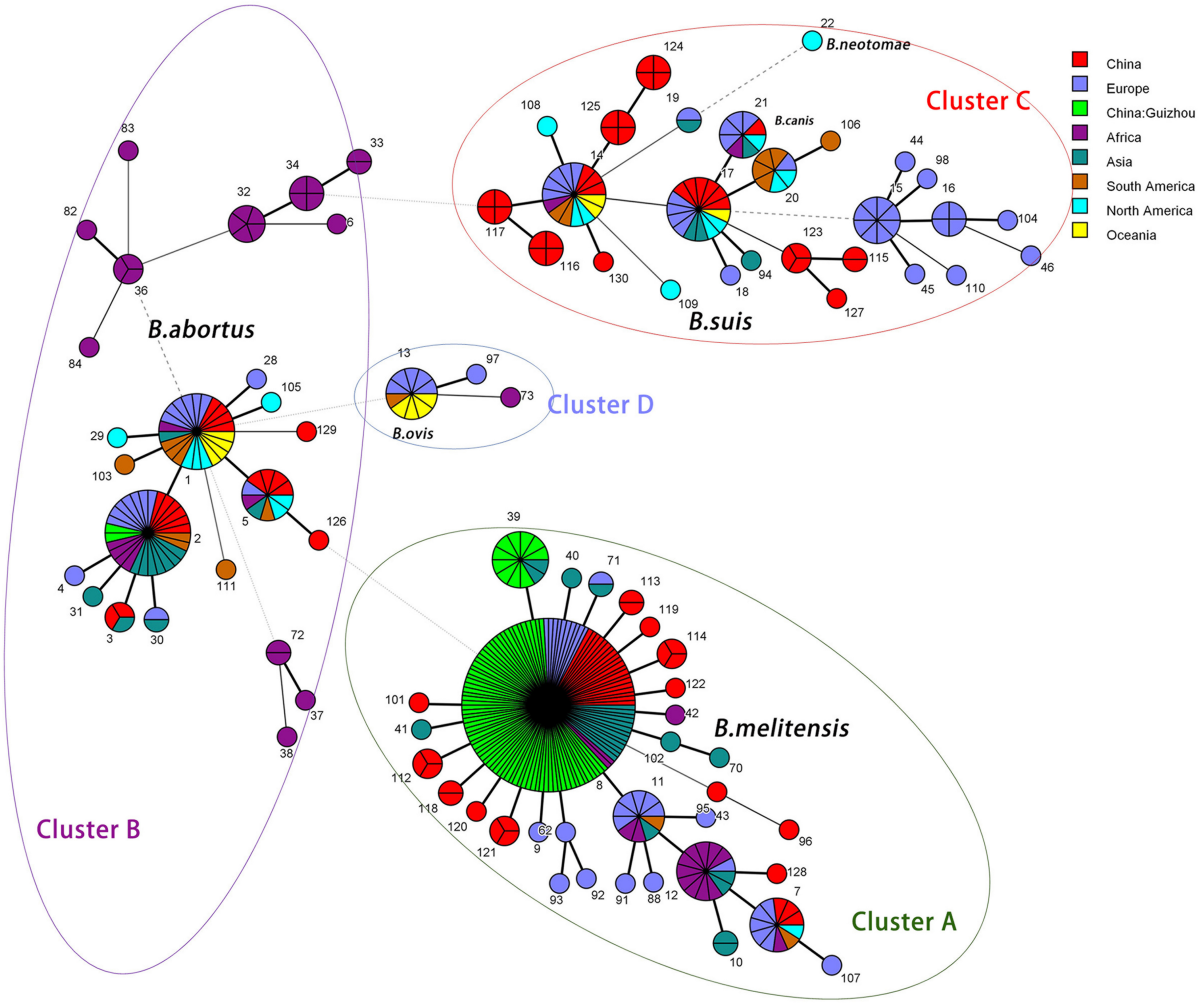
The clustering analysis MST (minimum spanning tree for categorical data) is based on MLST data of 401 *Brucella* across the world. Cluster A is an aggregation of *B. melitensis*, cluster B is composed of *B. abortus*, cluster C is composed of *B. suis, B. canis*, and *B. neotomae* while cluster D is composed of *B. vois*. A node represents a sequence type (ST), and the number represents the ST name. The node size is related to the number of strains, the more strains the larger the node, the less strains the smaller the node. The thickness of the line between the nodes is related to the genotypic affinity, with thicker and shorter lines representing closer affinity and thinner and longer lines representing more distant affinity.

**Figure 4:**
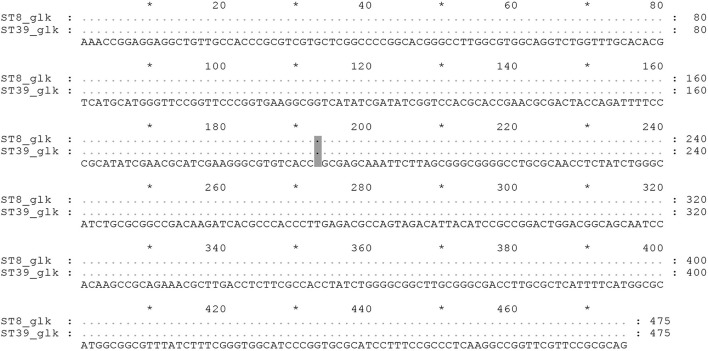
The *glk* gene sequence comparison between ST8 and ST39.

The MST showed that the *Brucella* strains of ST8 are mainly distributed in Asia, Europe, and Africa, while the ST39 genotype strains are distributed only in Asia, including Guizhou Province, China (Figure 3). A close genetic distance between the genotype ST39 and ST8 of *Brucella* strains showed in the MST, and the allelic profiles also showed their differences only at the housekeeping gene *glk* (Table 3). Subsequently, the *glk* gene sequence comparison between ST8 and ST39 differed at only one base site (Figure 4).

### MLVA genotyping results

The fragment sizes of the VNTR of 16 loci of the 83 isolates were successfully obtained by sequencing and multicolor capillary electrophoresis. The 81 *B. melitensis*, the VNTR value, and the Hunter and Gaston diversity indexes of 16 loci were calculated (Table 4). Specifically, the four loci in panel 2B (Bruce 04, 09, 16, and 30) showed relatively high discriminatory power, with the HGDI values ranging from 0.722 to 0.756, while another seven loci (Bruce 06, 08, 11, 18, 19, 21, and 55) displayed only a single allele (HGDI = 0.000). For the 2 *B. abortus* strains, the Hunter and Gaston diversity indexes at each locus were 0.000, which means the 16 loci lacked discrimination between them.

**Table 4:** The HGDI of the 16 loci among the 81 *B. melitensis* isolates.

**VNTR locus**	**Number of tandem repeat sequence locus**															HGDI^*^
	**1**	**2**	**3**	**4**	**5**	**6**	**7**	**8**	**9**	**10**	**11**	**12**	**13**	**14**	**41**	
Bruce 06	81	0	0	0	0	0	0	0	0	0	0	0	0	0	0	0.000
Bruce 08	0	0	0	0	81	0	0	0	0	0	0	0	0	0	0	0.000
Bruce 11	0	0	81	0	0	0	0	0	0	0	0	0	0	0	0	0.000
Bruce 12	0	0	0	0	0	0	0	0	0	0	0	2	79	0	0	0.049
Bruce 42	0	48	33	0	0	0	0	0	0	0	0	0	0	0	0	0.489
Bruce 43	6	75	0	0	0	0	0	0	0	0	0	0	0	0	0	0.139
Bruce 45	0	3	78	0	0	0	0	0	0	0	0	0	0	0	0	0.072
Bruce 55	0	81	0	0	0	0	0	0	0	0	0	0	0	0	0	0.000
Bruce 18	0	0	0	81	0	0	0	0	0	0	0	0	0	0	0	0.000
Bruce 19	0	0	0	0	0	0	0	0	0	0	0	0	0	0	81	0.000
Bruce 21	0	0	0	0	0	0	0	81	0	0	0	0	0	0	0	0.000
Bruce 04	0	0	2	35	13	13	8	8	2	0	0	0	0	0	0	0.750
Bruce 07	0	0	0	77	4	0	0	0	0	0	0	0	0	0	0	0.095
Bruce 09	0	0	37	2	1	3	6	11	6	8	4	0	2	1	0	0.756
Bruce 16	0	0	2	36	17	15	8	2	0	1	0	0	0	0	0	0.722
Bruce 30	0	0	8	27	23	19	2	2	0	0	0	0	0	0	0	0.752

^*^HGDI, Hunter and Gaston diversity index.

The 83 *Brucella* isolates, generated 49 MLVA genotypes based on the MLVA-16 profile, 33 of which were possessed by a single strain, and the other 16 genotypes were shared by two or more *Brucella* strains. The MLVA-11 profile of the 83 *Brucella* generated five known MLVA genotypes: 116, 125, 120, 115, and 72, and two unreported genotypes (NA1 and NA2). The MLVA-11 profiles showed that NA1 is different from genotype 120 at Bruce 12 with only one repeat-unit difference. Similarly, NA2 differs from genotype 116 only at Bruce 45 (Table 5).

**Table 5:** The MLVA-11 genotypes characteristics of the 83 *Brucella* strains isolates.

**MLVA-11 type**	Profile of MLVA-11^*^	**Species**	**No. of strain**	**Percentage**
116	1-5-3-13-2-2-3-2-4-41-8	*B. melitensis*	44	53.01%
125	1-5-3-13-3-2-3-2-4-41-8	*B. melitensis*	27	32.53%
120	1-5-3-13-3-1-3-2-4-41-8	*B. melitensis*	5	6.02%
115	1-5-3-12-2-2-3-2-4-41-8	*B. melitensis*	1	1.21%
NA1	1-5-3-12-3-1-3-2-4-41-8	*B. melitensis*	1	1.21%
NA2	1-5-3-13-2-2-2-2-4-41-8	*B. melitensis*	3	3.61%
72	4-5-3-12-2-2-3-1-6-43-8	*B. abortus*	2	2.41%

^*^The order of the MLVA-11 loci profiles is bruce 06, 08, 11, 12, 42, 43, 45, 55, 18, 19, and 21.

**Figure 5:**
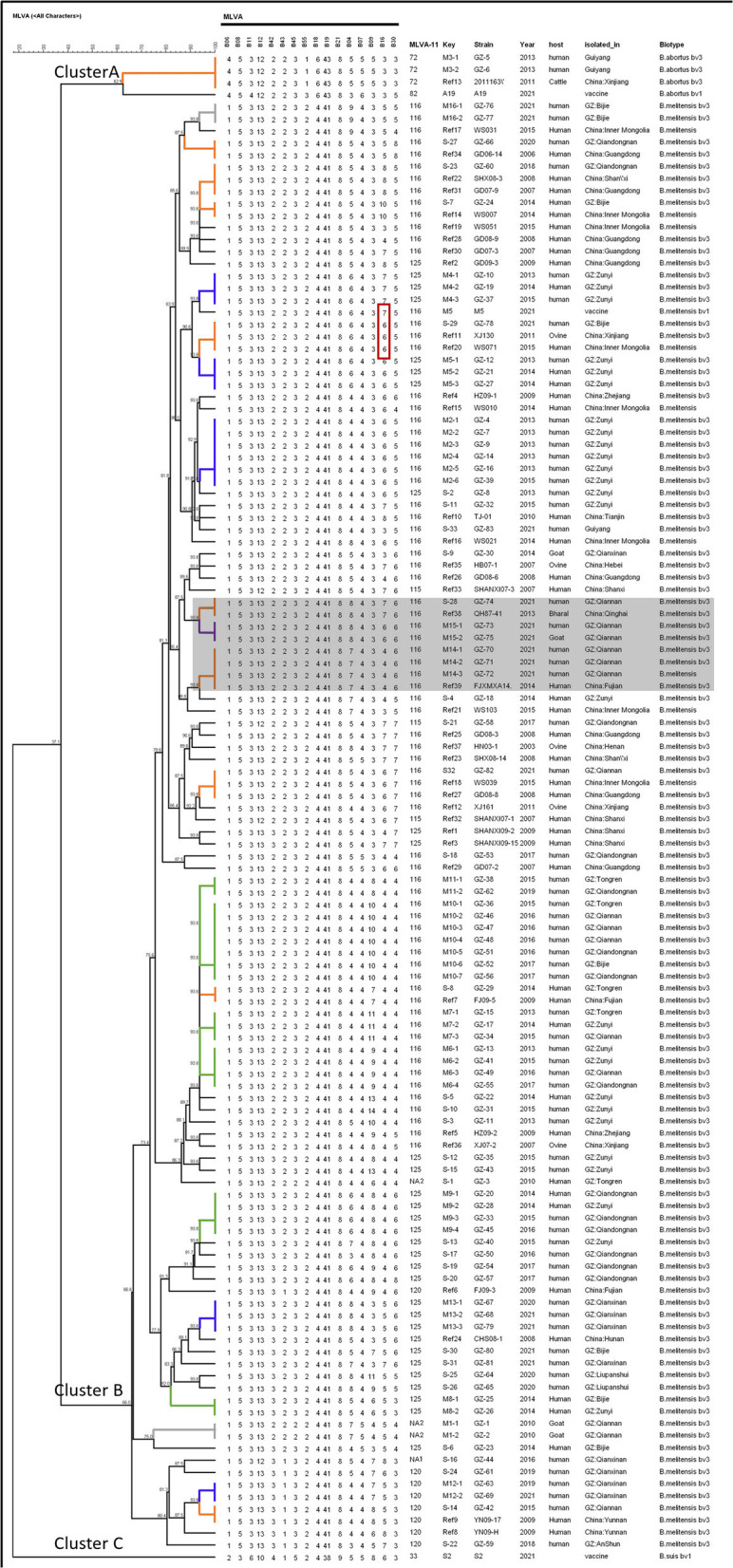
Dendrogram based on the MLVA-16 profile (UPGMA method) shows the correlation between 83 *Brucella* strains in Guizhou Province, 3 vaccine strains, and 39 representative strains of *Brucella* from different parts of the country. The columns show the MLVA-16 genotypes profile, MLVA-11 (panels 1 and 2A) genotypes, identification numbers, the geographic location, the year of isolation, and species-biovar of the strains. In the key column, M and S are the shared genotype and single genotype codes of *Brucella* in Guizhou Province, respectively. The purple branches represent shared genotypes from different hosts. Gray branches represent shared genotypes from the same region during the same period; blue branches represent shared genotypes from the same region but did differ in the period of isolation; green branches represent shared genotypes from cross-regional distribution, and also, in part, a cross-year distribution; black branch represents monogenic *Brucella*. The orange branch shows the shared genotype between the Guizhou Province isolates and the domestic strains.

The clustering analysis showed that the 125 *Brucella* strains, including the 83 isolates, 3 vaccine strains, and 39 representative strains of *Brucella* from different parts of China, formed three clusters (Figure 5). In Cluster A, the 2 *B. abortus* strains shared a genotype with the *B. abortus* bv3 from Xingjiang and clustered together with vaccine strain A19. In Cluster B, the 119 *B. melitensis* strains clustered together with *B. melitensis* vaccine M5. The vaccine S2 is a separate group. On the branches of the tree, the two strains GZ-73 and GZ-75 of the purple branch shared a genotype and were isolated from a human and a goat in the same outbreak, respectively. The *Brucella* strains of shared genotypes on the green branches, which has a cross-regional and, to some extent, cross-year distribution. The *Brucella* strains of shared genotypes on the blue branches were from the same region but differ in time. The *Brucella* strains of shared genotype on the gray and two orange branches (M3 and M14 in the key column) were from the same region during the same period. In addition, the *Brucella* strains of shared genotypes on the orange branches showed that Guizhou shared three genotypes with both Inner Mongolia and Guangdong, followed by two shared genotypes with Xinjiang and Fujian, and one shared genotype with Qinghai, Shaanxi, and Yunnan. Finally, it can be seen that the vaccine M5 differs from the *Brucella* GZ-78 and the *Brucella* from Xinjiang and Inner Mongolia in a duplicated fragment at Bruce 16 (Figure 5).

**Figure 6:**
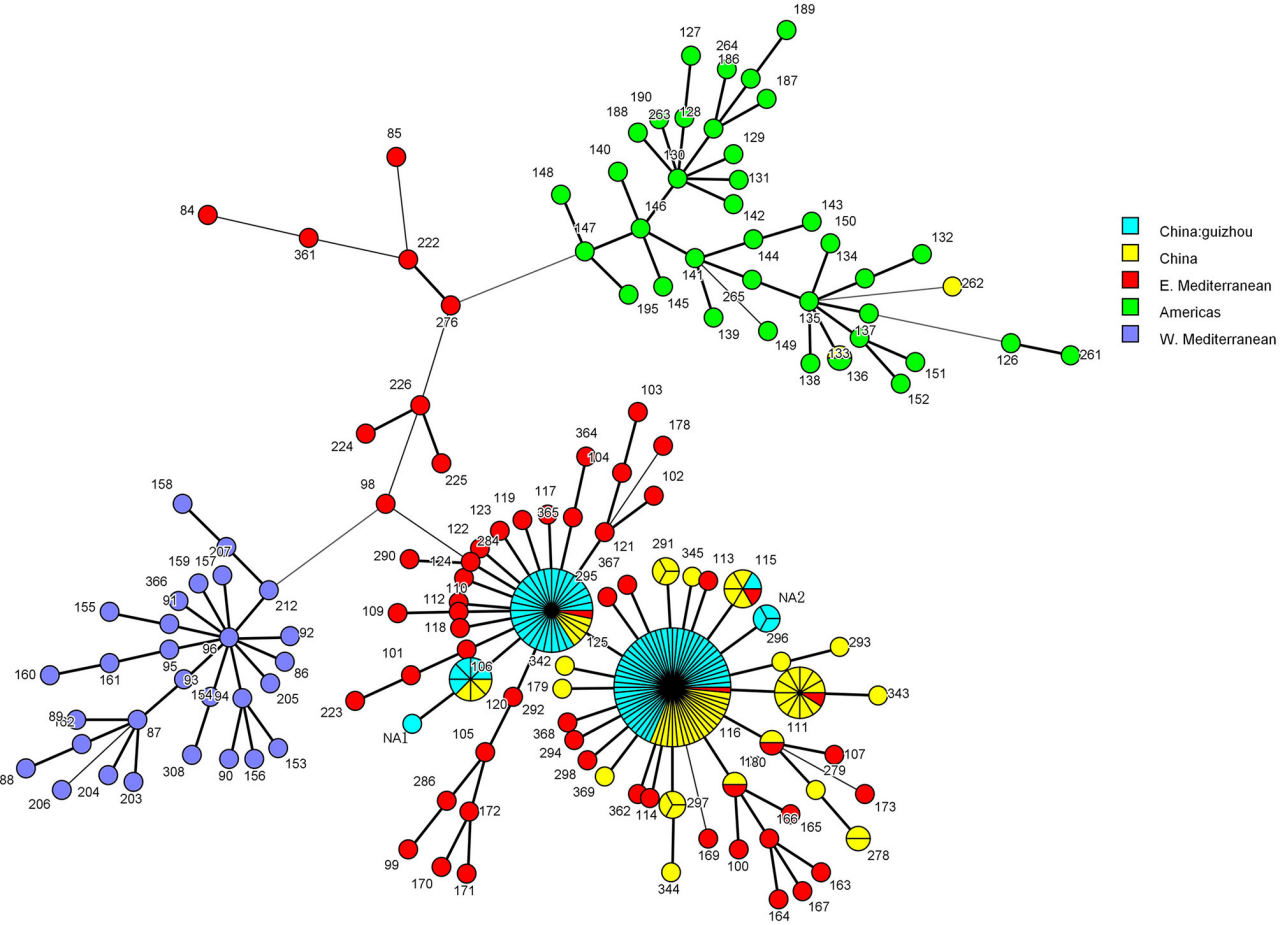
The MST of 269 *B. melitensis* strains based on the MLVA-11 profiles. colors of the nodes correspond to the Geographical distribution of *Brucella* strains: Eastern Mediterranean group (red), American group (green), Western Mediterranean group (purple), Chinese group (yellow), and the Guizhou isolate of this study (blue). A node represents an MLVA-11 genotype, and the number represents the genotype name. The node size is related to the number of strains; the more the number of strains the larger the node, the less number the smaller the node. The thickness of the line between the nodes is related to the genotypic affinity, with thicker and shorter lines representing closer affinity and thinner and longer lines representing more distant affinity.

Based on MLVA-11 profiles, a total of 401 *Brucella*, including 83 *Brucella* from Guizhou Province and 318 *Brucella* from Domestic and foreign, were defined as 87 MLVA-11 genotypes. The MST showed that the genotype NA1 and NA2 had the closest genetic distance to the genotypes 120 and 116, respectively, and all of the *B. melitensis* isolates from Guizhou Province are the members of the “East Mediterranean” group (Figure 6). Genotype 116, the predominant genotype, was broadly distributed in Guizhou Province (Figures 1, 6). The 2 *B. abortus*, the MLVA-11 genotype 72, also belonged to the “East Mediterranean” group.

### The *rpoB* genotyping results

A total of 83 *Brucella* isolates, using the *rpoB* gene sequence of *B. melitensis* 16M as a reference, identified six *rpoB* genotypes, including five *rpoB* genotypes of *B. melitensis* and one *rpoB* genotype of *B. abortus*. The 81 *B. melitensis* isolates generated 5 *rpoB* genotypes. Specifically, five Nucleotide polymorphisms were observed, two of which were new compared to previous studies (Tan et al., 2017), one at locus 582 involving a C to T substitution and the other at locus 1,135 involving a G to A substitution. Compared to the *rpoB* sequence of *B. melitensis* 16M, the *Brucella* isolates, involving C to T substitutions only at the 3,927 locus, which was named *B. melitensis* IIa1 in this study. A sequence based on IIa1, an additional variant locus 1,135, involving G to A substitution, was named *B. melitensis* IIa2, and an additional variant locus 1,886, involving C to T substitution was named *B. melitensis* IIb. Differ from the sequence of IIb, an additional variant locus 2,954, involving C to T substitution, which was named *B. melitensis* IIc. Finally, *B. melitensis* IId has an additional variant at locus 582 on the sequence of IIc (Table 6). The *B. melitensis* IIc was abundant in number, and widely distributed in eight cities/prefectures in the Province, except for Anshun (Figure 1).

**Table 6:** The 5 *rpoB* genotypes characteristics of the 81 *B. melitensis* isolates.

***rpoB* genotype**	**Single nucleotide polymorphisms profile** ^*^					**No. of strains**	**Percentage**
	**582**	**1,135**	**1,886**	**2,954**	**3,927**		
16M	C	G	C	C	G	/	/
IIa1	-	-	-	-	A	2	2.47%
IIa2	-	A	-	-	A	6	7.41%
IIb	-	-	T	-	A	11	13.58%
IIc	-	-	T	T	A	61	75.31%
IId	T	-	T	T	A	1	1.23%

^*^Nucleotide loci polymorphisms profile are observed based on the reference sequence of 16M—represents the base identity of the locus with the reference sequence.

**Figure 7:**
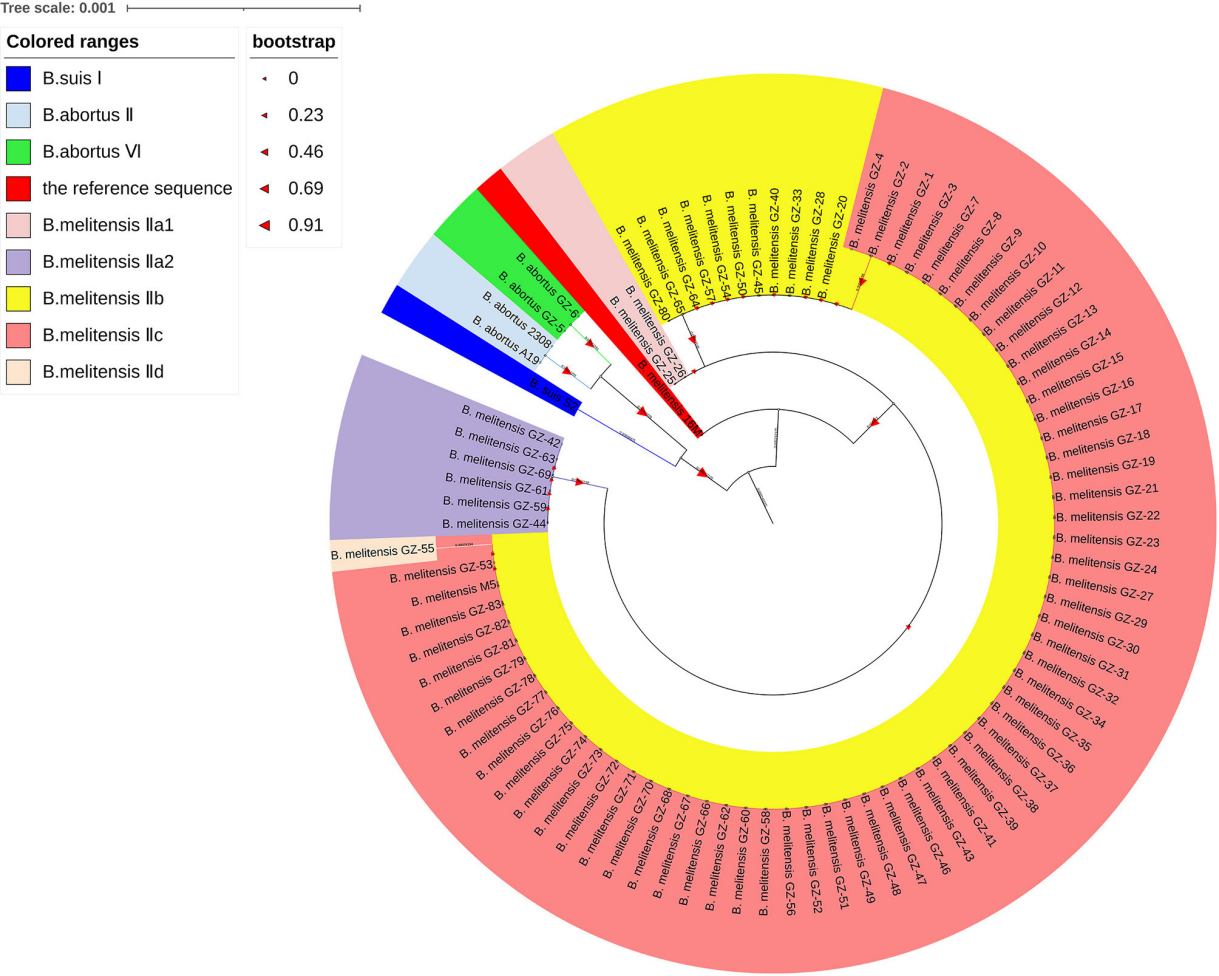
The Phylogenetic analysis of the 88 *Brucella* strains. Each color corresponds to one *rpoB* genotype, dark blue represents *rpoB* genotype *B. suis* I, blue represents *rpoB* genotype *B. abortus* II, green represents *rpoB* genotype *B. abortus* VI, red represents *rpoB* genotype of the reference sequence, pink represents *rpoB* genotype *B. melitensis* IIa1, purple represents *rpoB* genotype *B. melitensis* IIa2, dark yellow represents *rpoB* genotype *B. melitensis* IIb, orange-pink represents *rpoB* genotype *B. melitensis* IIc, and light orange represents *rpoB* genotype *B. melitensis* IId.

The phylogenetic analysis of the 83 *Brucella* isolates, based on the *rpoB* gene, revealed a similar relationship to that proposed above. The 81 *B. melitensis* on the same evolutionary branch. The *B. melitensis* 16M was at the front of the evolutionary branch, and *B. melitensis* IIa1 evolved from it, then both the IIa2 and IIb evolved from IIa1, and then, IIc evolved from IIb and IId evolved from IIc (Figure 7).

Five Nucleotide polymorphisms were observed in the 2 *B. abortus* (729, T to C; 804, G to T; 1,020, G to A; 2,211, T to C; 2,907, C to T). They were defined as *B. abortus* VI according to Marianelli C′ study (Marianelli et al., 2006). The *rpoB* genotypes of the three vaccine strains were defined according to their single nucleotide polymorphism loci concerning the study of Marianelli C' (Table 7) (12). Specifically, the *rpoB* genotype of M5, A19, and S2 is *B. melitensis* IIc, *B. abortus* II, and *B. suis* I, respectively. The phylogenetic analysis showed that the *B. suis* and the *B. abortus* belong to the same evolutionary branch and have a closer relationship (Figure 7).

**Table 7:** The *rpoB* genotypes characteristics of the 2 *B. abortus* isolates, *B. abortus* 2,308, vaccine A19 and S2.

**Strain**	***rpoB* genotype**	**Single nucleotide polymorphisms profile** ^*^							
		**729**	**804**	**812**	**1,020**	**2,148**	**2,211**	**2,907**	**3,603**
16M	*B. melitensis* III	T	G	T	G	G	T	C	C
*B. abortus GZ-5*	*B. abortus* VI	C	T	-	A	-	C	T	-
*B. abortus* GZ-6	*B. abortus* VI	C	T	-	A	-	C	T	-
Vaccine A19	*B. abortus* II	C	T	-	-	A	C	T	-
*B. abortus* 2308	*B. abortus* II	C	T	-	-	A	C	T	-
Vaccine S2	*B. suis* I	C	-	C	-	-	-	T	T

^*^Nucleotide loci polymorphisms profile are observed based on the reference sequence of 16M—represents the base identity of the locus with the reference sequence.

## Discussion

In this study, three typing methods were applied to identify *Brucella* species in Guizhou Province. Although there were some incongruences in genotypes, all methods showed a consistent conclusion that there are two *Brucella* species in the sample analyzed. In Guizhou Province, *B. meltenesis* is the dominant species and infected goats are the main infection source of human brucellosis here. Meanwhile, the 2 *B. abortus* strains were isolated from two dairy farm veterinarians suggesting that cows are also the source of human brucellosis infection. The *B. abortus* is also a potential threat to Guizhou Province. The first *B. melitensis* of Guizhou Province was isolated in Qiannan Buyi and Miao Autonomous Prefecture in 2010 (Supplementary Table S1), and the highest number of human brucellosis cases was in Zunyi City, followed by Tongren and Bijie (Figure 1). All these areas have one thing in common is that they all share borders with neighboring provinces and cities, which provides a clue to the extra-provincial importation of the epidemic in Guizhou Province, and the shared genotype of MLVA-16 of *Brucella* in Guizhou and other places in China makes the inference of extra-provincial importation confirmed. It is worth noting that there are not many human brucelloses in Qianxinan Buyi and Miao Autonomous Prefecture, but its genotypes are the most abundant in Guizhou Province. It indicates that livestock in the region may be small-scale farming, reducing the risk of large-scale transmission of brucellosis. It may be possible that most local farmers are not aware of brucellosis which causes the importation of the disease. Epidemiological surveys indicate that human brucellosis in Guizhou Province is concentrated in the group of farmers aged 41–50 years, mainly due to their direct contact with infected goats or cows. Therefore, the relevant authorities should strengthen the publicity and education of high-risk groups who keep goats or cows to improve their awareness of personal protection and reduce their brucellosis infection rate.

ST8 is the main prevalent genotype in Guizhou Province and China (Ma et al., 2016; Tian et al., 2017b; Cao et al., 2018b). It is widely distributed in Asia, Europe, and Africa, and it is also a popular genotype in foreign countries (Shome et al., 2016; Akar and Erganis, 2022; Dadar et al., 2022). ST2 of *B. abortus* in Guizhou Province is also the genotype of *B. abortus* widely epidemic in China (Piao et al., 2018). These findings reveal that the epidemiology patterns of brucellosis in Guizhou Province were compatible with domestic. In addition to that the ST39 was the newly reported genotype in China, which suggests that the epidemiology of brucellosis in this region may be distinctive. The first *B. melitensis* of ST39 was isolated in Zunyi City in 2013, and in the following 2 years, the *Brucella* of this genotype also has been isolated in the city. And then, 7 years later, in 2020–2021, the ST39 strains were isolated again in Qianxinan Buyi and Miao Autonomous Prefecture. Epidemiological investigations showed that the isolates with ST39 in Qianxinan Buyi and Miao Autonomous Prefecture were associated with mating activities but could not be traced back to a transmission chain associated with Zunyi City. This may be due to the failure to isolate the strains for various reasons or the presence of potential infection in some animals that were not detected. The polymorphism of one nucleotide locus of the *glk* gene between ST39 and ST8 (Figure 5) and the MST clustering (Figure 4) further confirmed their close relationship. Furthermore, the widespread prevalence of ST8 *Brucella* strains in Guizhou Province suggests that the ST39 type may be derived from the ST8 mutation. Therefore, the focus needs to be on the origin of the type.

Among the 81 *B. melitensis* strains in Guizhou Province, Hunter and Gaston's diversity indexes revealed that panel 2B loci (Bruce 04, 09, 16, and 30) of MLVA had a high discriminatory power in them. Interestingly, the six isolates from an aggregated epidemic were identified as three genotypes, and differences in the three genotypes were at loci 04 and 16 (Tan et al., 2022a). Coincidentally, the differences in three genotypes of the five *Brucella* strains, isolated in an aggregated epidemic reported by Tian Guozhong, were also at loci 04 and 16 (Tian et al., 2017a). Furthermore, Bruce 04 and 16 have been reported to be highly polymorphic in many studies (Al Dahouk et al., 2007; Sun et al., 2016; Liu et al., 2017). Although the Bruce 16 locus was included in the mutational events observed by Whatmore et al., no mutations were observed in both *in vivo* and *in vitro* tests. The results of *in vivo* tests on pigs may be biased considering that pigs are not the dominant host for *B. melitensis* (Whatmore et al., 2006). Therefore, differences between *B. melitensis* at loci 04 and 16 should not be used as a basis for excluding an epidemic association when using the MLVA technique for molecular epidemiological investigations of brucellosis outbreaks. In addition, the similarity among the three MLVA-16 genotypes in this aggregated outbreak in Guizhou Province was 85.4% (shaded area in Figure 5), and the data evidence that all the strains originated from the same outbreak are poor. However, the *six Brucella* strains all belonged to ST8 and *rpoB* IIc (Supplementary Table S1), which confirmed the close kinship between the isolates. So, combining MLST and *rpoB* typing methods for epidemiologic tracing can avoid erroneous judgments, which is essential for epidemiological traceability analysis. In this study, the difference between vaccine M5 and *Brucella* GZ-78 with only one repeat fragment at Bruce 16 was observed. Cases of brucellosis caused by vaccine strains do exist (Baoshan et al., 2021). It cannot be excluded whether the infection was caused by live vaccine strain M5. Guizhou Province is classified as a category II area by the Ministry of Agriculture and the National Health and Family Planning Commission, so a non-immune decontamination and surveillance strategy among animals is also the best choice for brucellosis prevention and control in Guizhou Province.

Forty-nine genotypes were generated based on the profile of 16 VNTR loci in the 83 *Brucella* isolates, 33 of which were possessed by a single strain, suggesting that they were derived from sporadic or unlinked epidemiological cases, revealing a scattered epidemic of brucellosis. The other 16 MLVA-16 shared genotypes were shared by 2 and more strains, suggesting a potential molecular epidemiological link between these strains and a possible aggregated outbreak involving multiple regions. The two strains of shared genotypes M15 from different hosts revealed the infection source of the patient from the molecular epidemiological perspective. The strains of shared genotypes, from the same area during the same period suggest the existence of an aggregated brucellosis outbreak in the area, while different regions during the same period suggest the presence of transit or circulation of infected animals and their products in different regions. Most of the *Brucella* strains of the shared genotype were isolated at Zunyi City and were found earlier (Figure 5), which may be related to the better medical conditions in Zunyi City. Also, the shared genotypic *Brucella* strains from different cities/states in the province revealed the intra-provincial spread of the brucellosis epidemic. The shared genotypes of *Brucella* strains between Guizhou and Inner Mongolia, Xinjiang, Guangdong, Fujian, Qinghai, Shaanxi, and Yunnan, reveal the existence of the transfer and circulation of infected animals and their products between Guizhou Province and the aforementioned regions. Meanwhile, the results suggest that there is an out-of-province import of the brucellosis epidemic in Guizhou Province. Currently, the brucellosis-affected areas have expanded from northern pastureland provinces to southern coastal and southwestern areas (Zheng et al., 2018). So, Guizhou Province should strictly control the inspection and quarantine of animals across the region.

All of the *Brucella* isolates in Guizhou Province are members of the “East Mediterranean” group (Zhu et al., 2020), revealing the geographic origin of *Brucella* in Guizhou Province. The predominant genotype 116 of MLVA-11 genotypes of *Brucella* in Guizhou Province was consistent in most provinces of China (Liu et al., 2017, 2019, 2020; Tian et al., 2017b; Cao et al., 2018a; Li et al., 2020). Interestingly, the genotype 116 is also the predominant genotype of *Brucella* that continues to spread and expand from northern to southern China (Zhu et al., 2020). These findings confirm the out-of-province import of *Brucella* in Guizhou Province, which is consistent with the results confirmed by the shared genotype of MLVA-16. Genotype 72 of MLVA-11 of *B. abortus* in Guizhou Province shared the same genotype as Xinjiang strains (Figure 5) and was consistent with the genotype circulating in Inner Mongolia (Ma et al., 2020), suggesting a possible geographical origin of *Brucella*. This inference is also fully in line with the migration route of *Brucella* in China (Tian et al., 2017b; Zhu et al., 2020), which has high application value for the tracing of the epidemic. Of the two unreported genotypes, NA1 is different from genotype 120 at Bruce 12 with only one repeat-unit difference and NA2 differs from genotype 116 only at Bruce 45. Meanwhile, The MST showed that the genotype NA1 and NA2 had the closest genetic distance to the genotypes 120 and 116, respectively. These findings reveal the possible origin of the new genotypes. This inference is supported by combining the MLST and *rpoB* typing results of these strains.

Based on the complete *rpoB* gene sequence, the evolutionary analysis revealed the affinities and possible evolutionary relationships among these strains. All *B. melitensis* strains on an evolutionary branch, while *B. suis* and *B. abortus* belong to the other evolutionary branch, which revealed that *B. melitensis* is more distantly related to *B. abortus* and *B. suis*, while the relationship between *B. abortus* and *B. suis* is even closer. This result deviates from the close kinship between *B. melitensis* and *B. abortus* based on whole-genome sequencing analysis (Suárez-Esquivel et al., 2020; Abdel-Glil et al., 2022). Probably because of the small number of *Brucella* species in this study and the high interspecific DNA homology of bacteria of the genus *Brucella* (Hoyer and McCullough, 1968; Ficht, 2010; Suárez-Esquivel et al., 2020), the discriminatory power of the *rpoB* gene in the evolutionary analysis between *Brucella* species was slightly poor. However, the evolutionary analysis of the *rpoB* gene reveal the relationship between the various types of bacteria within *Brucella* species. The evolutionary relationship of the five *rpoB* genotypes of *B. melitensis* isolates in the upper and lower branches of the evolutionary tree further indicates the close kinship among *B. melitensis* in Guizhou Province, and also provides a strong laboratory basis for the provincial spread of the brucellosis epidemic. The evolutionary analysis of interspecific *rpoB* genes within the genus *Brucella* needs to be treated with caution. However, the evolutionary relationships within species can be presented, which contributes to the analysis of the traceability and evolutionary relationships among the intra-species bacteria.

The three typing techniques were consistent at the level of *Brucella* species discrimination, but to the gene type, MLVA had the highest resolution, the *ropB* genotype the second, and MLST the least (Supplementary Table S1). The 2 *B. abortus* strains were indistinguishable by three techniques, suggesting an origin from the same source of infection. By combining the three typing methods, all the *Brucella* strains of MLVA-11 type 116 belonged to the ST8, while producing IIc and IId by *rpoB* typing. The evolutionary analysis showed that IId was derived from the IIc mutation which the codon was synonymously mutated from ATC to ATT, but did not cause the amino acid change. Similarly, the six IIa2 genotype strains belonged to the ST8, while producing type 120 and NA1 by MLVA-11 typing, indicating a close affinity between the two types. It was also demonstrated by MST analysis (Figure 6). Therefore, the combination of three typing techniques can better analyze the new genotype strains and trace the strains. In addition, although the MLVA typing technique has a high resolution, differences in highly polymorphic loci are prone to misclassification, a combination of MLST with *rpoB* typing methods would be beneficial for the tracking and analysis of the epidemic. Therefore, the three techniques are combined and complement each other and can be better used for traceability analysis of epidemics.

## Conclusion

The *Brucella* isolates in Guizhou Province are closely related to each other. Also, they have close affinities with *Brucella* in various parts of China. The brucellosis epidemic in Guizhou Province has both an intra-provincial spread and extra-provincial import transmission. Given that the majority of brucellosis-infected persons are among its high-risk groups, it is important that the relevant authorities strengthen the awareness and education of high-risk groups. Meanwhile, in the introduction of livestock, trade, and the circulation of its products implement strict inspection and quarantine. In short, the prevention and control of brucellosis in Guizhou Province require strong, multi-sectoral joint prevention and control.

The MLVA typing technique has a unique advantage in the traceability analysis of brucellosis epidemics due to its high resolution, but the difference at loci 04 and 16 cannot exclude the association between *Brucella*. So, combining MLST and *rpoB* typing methods for traceability analysis of outbreaks is of great importance. Moreover, by combining the three typing techniques, the possible origin of the new genotype *Brucella* can be reasonably inferred, which is also conducive to promoting the subsequent research of the novel bacteria.

## Data availability statement

The original contributions presented in the study are included in the article/Supplementary material, further inquiries can be directed to the corresponding authors.

## Author contributions

QT, YW, and SL designed the study. ZT, CY, and YHua collected the samples and epidemiological data. QT, XYa, XYi, and YHu analyzed the data. QT drafted the manuscript. SL revised the manuscript. All authors contributed to the article and approved the submitted version.
