# Supplementary material for: Molecular epidemiological characteristics of Brucella in Guizhou Province, China, from 2009 to 2021
Source: Front Microbiol. 2023 Jun 23;14:1188469. doi: 10.3389/fmicb.2023.1188469 (PMC10326899; doi:10.3389/fmicb.2023.1188469)
Supplement: Supplementary file 1 [file Table_1.docx]

Supplementary Material

**Molecular Epidemiological Characteristics of *Brucella* in Guizhou Province, China, during 2009-2021**

**Qinqin Tan^1,2†^, Yue Wang^1†^, Ying Liu^1^, Zhongfa Tao^3^, Chun Yu^3^, Yan Huang^3^, Xinggui Yang^1^, Xia Ying^1,2^,** **Yong Hu^2^**^*^**，Shijun Li^1,2^**^*^

^1^Laboratory Center, Center for Disease Control and Prevention of Guizhou Provincial, Guiyang, Guizhou, China

^2^The Key Laboratory of Environmental Pollution Monitoring and Disease Control, Ministry of Education, School of Public Health, University of Guizhou Medical, Guiyang, Guizhou, China

^3^Institute of Infectious Disease Control, Center for Disease Control and Prevention of Guizhou Provincial, Guiyang, Guizhou, China

**Equal contributions:**

^†^These authors contributed equally to this work and share first authorship.

*** Correspondence:**

Shijun Li*

zjumedjun@163.com

Yong Hu*

huyong1979@gmc.edu.cn

# Supplementary Tables

**Table S1.** **Molecular typing results of the 83 *Brucella* isolates in Guizhou**

| No. | strain | year | isolated_in | host | MLVA-11 | MLVA-16 | ST | rpoB |
| --- | --- | --- | --- | --- | --- | --- | --- | --- |
| 1 | GZ-1 | 2010 | Qiannan Buyi and Miao Autonomous Prefecture | Goat | NA2 | M-1 | 8 | *B. melitensis* Ⅱc |
| 2 | GZ-2 | 2010 | Qiannan Buyi and Miao Autonomous Prefecture | Goat | NA2 | M-1 | 8 | *B. melitensis* Ⅱc |
| 3 | GZ-3 | 2011 | Tongren | Human | NA2 | / | 8 | *B. melitensis* Ⅱc |
| 4 | GZ-4 | 2013 | Zunyi | human | 116 | M-2 | 8 | *B. melitensis* Ⅱc |
| 5 | GZ-5 | 2013 | Guiyang | human | 72 | M-3 | 2 | *B. abortus* Ⅵ |
| 6 | GZ-6 | 2013 | Guiyang | human | 72 | M-3 | 2 | *B. abortus* Ⅵ |
| 7 | GZ-7 | 2013 | Zunyi | human | 116 | M-2 | 8 | *B. melitensis* Ⅱc |
| 8 | GZ-8 | 2013 | Zunyi | human | 125 | / | 8 | *B. melitensis* Ⅱc |
| 9 | GZ-9 | 2013 | Zunyi | human | 116 | M-2 | 8 | *B. melitensis* Ⅱc |
| 10 | GZ-10 | 2013 | Zunyi | human | 125 | M-4 | 39 | *B. melitensis* Ⅱc |
| 11 | GZ-11 | 2013 | Zunyi | human | 116 | / | 8 | *B. melitensis* Ⅱc |
| 12 | GZ-12 | 2013 | Zunyi | human | 125 | M-5 | 39 | *B. melitensis* Ⅱc |
| 13 | GZ-13 | 2013 | Zunyi | human | 116 | M-6 | 8 | *B. melitensis* Ⅱc |
| 14 | GZ-14 | 2013 | Zunyi | human | 116 | M-2 | 8 | *B. melitensis* Ⅱc |
| 15 | GZ-15 | 2013 | Tongren | human | 116 | M-7 | 8 | *B. melitensis* Ⅱc |
| 16 | GZ-16 | 2013 | Zunyi | human | 116 | M-2 | 8 | *B. melitensis* Ⅱc |
| 17 | GZ-17 | 2014 | Zunyi | Human | 116 | M-7 | 8 | *B. melitensis* Ⅱc |
| 18 | GZ-18 | 2014 | Zunyi | Human | 116 | / | 8 | *B. melitensis* Ⅱc |
| 19 | GZ-19 | 2014 | Zunyi | Human | 125 | M-4 | 39 | *B. melitensis* Ⅱc |
| 20 | GZ-20 | 2014 | Qiandongnan Miao and Dong Autonomous prefecture | Human | 125 | / | 8 | *B. melitensis* Ⅱb |
| 21 | GZ-21 | 2014 | Zunyi | Human | 125 | M-5 | 39 | *B. melitensis* Ⅱc |
| 22 | GZ-22 | 2014 | Zunyi | Human | 116 | / | 8 | *B. melitensis* Ⅱc |
| 23 | GZ-23 | 2014 | Bijie | Human | 125 | / | 8 | *B. melitensis* Ⅱc |
| 24 | GZ-24 | 2014 | Bijie | Human | 116 | / | 8 | *B. melitensis* Ⅱc |
| 25 | GZ-25 | 2014 | Bijie | Human | 125 | M-8 | 8 | *B. melitensis* Ⅱa1 |
| 26 | GZ-26 | 2014 | Zunyi | Human | 125 | M-8 | 8 | *B. melitensis* Ⅱa1 |
| 27 | GZ-27 | 2014 | Zunyi | Human | 125 | M-5 | 39 | *B. melitensis* Ⅱc |
| 28 | GZ-28 | 2014 | Zunyi | Human | 125 | / | 8 | *B. melitensis* Ⅱb |
| 29 | GZ-29 | 2014 | Tongren | Human | 116 | / | 8 | *B. melitensis* Ⅱc |
| 30 | GZ-30 | 2014 | Qianxinan Buyi and Miao Autonomous Prefecture | Goat | 116 | / | 8 | *B. melitensis* Ⅱc |
| 31 | GZ-31 | 2015 | Zunyi | human | 116 | / | 8 | *B. melitensis* Ⅱc |
| 32 | GZ-32 | 2015 | Zunyi | human | 116 | / | 8 | *B. melitensis* Ⅱc |
| 33 | GZ-33 | 2015 | Qiandongnan Miao and Dong Autonomous prefecture | human | 125 | M-9 | 8 | *B. melitensis* Ⅱb |
| 34 | GZ-34 | 2015 | Qiannan Buyi and Miao Autonomous Prefecture | human | 116 | M-7 | 8 | *B. melitensis* Ⅱc |
| 35 | GZ-35 | 2015 | Zunyi | human | 125 | / | 8 | *B. melitensis* Ⅱc |
| 36 | GZ-36 | 2015 | Tongren | human | 116 | M-10 | 8 | *B. melitensis* Ⅱc |
| 37 | GZ-37 | 2015 | Zunyi | human | 125 | M-4 | 39 | *B. melitensis* Ⅱc |
| 38 | GZ-38 | 2015 | Tongren | human | 116 | M-11 | 8 | *B. melitensis* Ⅱc |
| 39 | GZ-39 | 2015 | Zunyi | human | 116 | M-2 | 8 | *B. melitensis* Ⅱc |
| 40 | GZ-40 | 2015 | Zunyi | human | 125 | / | 8 | *B. melitensis* Ⅱb |
| 41 | GZ-41 | 2015 | Zunyi | human | 116 | M-6 | 8 | *B. melitensis* Ⅱc |
| 42 | GZ-42 | 2015 | Qiannan Buyi and Miao Autonomous Prefecture | human | 120 | / | 8 | *B. melitensis* Ⅱa2 |
| 43 | GZ-43 | 2015 | Zunyi | human | 125 | / | 8 | *B. melitensis* Ⅱc |
| 44 | GZ-44 | 2016 | Qianxinan Buyi and Miao Autonomous Prefecture | human | NA1 | / | 8 | *B. melitensis* Ⅱa2 |
| 45 | GZ-45 | 2016 | Qiandongnan Miao and Dong Autonomous prefecture | human | 125 | M-9 | 8 | *B. melitensis* Ⅱb |
| 46 | GZ-46 | 2016 | Qiannan Buyi and Miao Autonomous Prefecture | human | 116 | M-10 | 8 | *B. melitensis* Ⅱc |
| 47 | GZ-47 | 2016 | Qiannan Buyi and Miao Autonomous Prefecture | human | 116 | M-10 | 8 | *B. melitensis* Ⅱc |
| 48 | GZ-48 | 2016 | Qiannan Buyi and Miao Autonomous Prefecture | human | 116 | M-10 | 8 | *B. melitensis* Ⅱc |
| 49 | GZ-49 | 2016 | Qiannan Buyi and Miao Autonomous Prefecture | human | 116 | M-6 | 8 | *B. melitensis* Ⅱc |
| 50 | GZ-50 | 2016 | Qiandongnan Miao and Dong Autonomous prefecture | human | 125 | / | 8 | *B. melitensis* Ⅱb |
| 51 | GZ-51 | 2016 | Qiandongnan Miao and Dong Autonomous prefecture | human | 116 | M-10 | 8 | *B. melitensis* Ⅱc |
| 52 | GZ-52 | 2017 | Bijie | human | 116 | M-10 | 8 | *B. melitensis* Ⅱc |
| 53 | GZ-53 | 2017 | Qiandongnan Miao and Dong Autonomous prefecture | human | 116 | / | 8 | *B. melitensis* Ⅱc |
| 54 | GZ-54 | 2017 | Qiandongnan Miao and Dong Autonomous prefecture | human | 125 | / | 8 | *B. melitensis* Ⅱb |
| 55 | GZ-55 | 2017 | Qiandongnan Miao and Dong Autonomous prefecture | human | 116 | M-6 | 8 | *B. melitensis* Ⅱd |
| 56 | GZ-56 | 2017 | Qiandongnan Miao and Dong Autonomous prefecture | human | 116 | M-10 | 8 | *B. melitensis* Ⅱc |
| 57 | GZ-57 | 2017 | Qiandongnan Miao and Dong Autonomous prefecture | human | 125 | / | 8 | *B. melitensis* Ⅱb |
| 58 | GZ-58 | 2017 | Qiandongnan Miao and Dong Autonomous prefecture | human | 115 | / | 8 | *B. melitensis* Ⅱc |
| 59 | GZ-59 | 2018 | Anshun | human | 120 | / | 8 | *B. melitensis* Ⅱa2 |
| 60 | GZ-60 | 2018 | Qiandongnan Miao and Dong Autonomous prefecture | human | 116 | / | 8 | *B. melitensis* Ⅱc |
| 61 | GZ-61 | 2019 | Qianxinan Buyi and Miao Autonomous Prefecture | human | 120 | / | 8 | *B. melitensis* Ⅱa2 |
| 62 | GZ-62 | 2019 | Qiandongnan Miao and Dong Autonomous prefecture | human | 116 | M-11 | 8 | *B. melitensis* Ⅱc |
| 63 | GZ-63 | 2019 | Qianxinan Buyi and Miao Autonomous Prefecture | human | 120 | M-12 | 8 | *B. melitensis* Ⅱa2 |
| 64 | GZ-64 | 2020 | Liupanshui | human | 125 | / | 8 | *B. melitensis* Ⅱb |
| 65 | GZ-65 | 2020 | Liupanshui | human | 125 | / | 8 | *B. melitensis* Ⅱb |
| 66 | GZ-66 | 2020 | Qiandongnan Miao and Dong Autonomous prefecture | human | 116 | / | 8 | *B. melitensis* Ⅱc |
| 67 | GZ-67 | 2020 | Qianxinan Buyi and Miao Autonomous Prefecture | human | 125 | M-13 | 39 | *B. melitensis* Ⅱc |
| 68 | GZ-68 | 2021 | Qianxinan Buyi and Miao Autonomous Prefecture | human | 125 | M-13 | 39 | *B. melitensis* Ⅱc |
| 69 | GZ-69 | 2021 | Qianxinan Buyi and Miao Autonomous Prefecture | human | 120 | M-12 | 8 | *B. melitensis* Ⅱa2 |
| 70 | GZ-70 | 2021 | Qiannan Buyi and Miao Autonomous Prefecture | human | 116 | M-14 | 8 | *B. melitensis* Ⅱc |
| 71 | GZ-71 | 2021 | Qiannan Buyi and Miao Autonomous Prefecture | human | 116 | M-14 | 8 | *B. melitensis* Ⅱc |
| 72 | GZ-72 | 2021 | Qiannan Buyi and Miao Autonomous Prefecture | human | 116 | M-14 | 8 | *B. melitensis* Ⅱc |
| 73 | GZ-73 | 2021 | Qiannan Buyi and Miao Autonomous Prefecture | human | 116 | M-15 | 8 | *B. melitensis* Ⅱc |
| 74 | GZ-74 | 2021 | Qiannan Buyi and Miao Autonomous Prefecture | human | 116 | / | 8 | *B. melitensis* Ⅱc |
| 75 | GZ-75 | 2021 | Qiannan Buyi and Miao Autonomous Prefecture | Goat | 116 | M-15 | 8 | *B. melitensis* Ⅱc |
| 76 | GZ-76 | 2021 | Bijie | human | 116 | M-16 | 8 | *B. melitensis* Ⅱc |
| 77 | GZ-77 | 2021 | Bijie | human | 116 | M-16 | 8 | *B. melitensis* Ⅱc |
| 78 | GZ-78 | 2021 | Bijie | human | 116 | / | 8 | *B. melitensis* Ⅱc |
| 79 | GZ-79 | 2021 | Qianxinan Buyi and Miao Autonomous Prefecture | human | 125 | M-13 | 39 | *B. melitensis* Ⅱc |
| 80 | GZ-80 | 2021 | Bijie | human | 125 | / | 8 | *B. melitensis* Ⅱb |
| 81 | GZ-81 | 2021 | Qianxinan Buyi and Miao Autonomous Prefecture | human | 125 | / | 39 | *B. melitensis* Ⅱc |
| 82 | GZ-82 | 2021 | Qiannan Buyi and Miao Autonomous Prefecture | human | 116 | / | 8 | *B. melitensis* Ⅱc |
| 83 | GZ-83 | 2021 | Guiyang | human | 116 | / | 8 | *B. melitensis* Ⅱc |
